# Paternal Determinants in Preeclampsia

**DOI:** 10.3389/fphys.2018.01870

**Published:** 2019-01-07

**Authors:** Carlos Galaviz-Hernandez, Martha Sosa-Macias, Enrique Teran, Jose Elias Garcia-Ortiz, Blanca Patricia Lazalde-Ramos

**Affiliations:** ^1^Instituto Politécnico Nacional, CIIDIR-Durango, Academia de Grnómica, Mexico City, Mexico; ^2^Colegio de Ciencias de la Salud, Universidad San Francisco de Quito, Quito, Ecuador; ^3^Centro de Investigacón Biomédica de Occidente, Centro Médico Nacional de Occidente-Instituto Mexicano del Seguro Social (CMNO-IMSS), Guadalajara, Mexico; ^4^Unidad Académica de Ciencias Químicas, Universidad Autónoma de Zacatecas, Zacatecas, Mexico

**Keywords:** preeclampsia, paternal, primipaternity, placenta, immunology, genetics

## Abstract

Preeclampsia is a condition associated with high rates of maternal-fetal morbidity and mortality. It usually occurs in 3–10% of nulliparous women and 18% of previously affected women. Different lines of evidence have demonstrated the role of the father in the onset of preeclampsia. The placenta is the cornerstone of preeclampsia and poses important paternal genetic determinants; in fact, the existence of a “paternal antigen” has been proposed. Nulliparity is a well-known risk factor. Change of partner to a woman without history of preeclampsia increases the risk; however, this change decreases in women with history of the condition. High interval between pregnancies, short sexual intercourse before pregnancy, and conception by intracytoplasmic sperm injection suggest a limited exposure to the so-called paternal antigen. A man who was born from a mother with preeclampsia also increases the risk to his partner. Not only maternal but also paternal obesity is a risk factor for preeclampsia. Fetal HLA-G variants from the father increased the immune incompatibility with the mother and are also significantly associated with preeclampsia in multigravida pregnancies. An analysis of a group of Swedish pregnant women showed that the risk for preeclampsia is attributable to paternal factors in 13% of cases, which could be related to genetic interactions with maternal genetic factors. This review aimed to evaluate the evidences of the father’s contribution to the onset of preeclampsia and determine the importance of including them in future studies.

## Background

Preeclampsia is a condition in pregnant women associated with high rates of maternal and fetal morbidity and mortality worldwide. This disease is characterized as a systemic syndrome with *de novo* hypertension occurring after 20 weeks of gestation as well as proteinuria (300 mg in 24 h) (NHBPEP, 2000). In 2013, the Task Force on Hypertension in Pregnancy established new diagnostic criteria for hypertension in pregnancy.

Preeclampsia is related to deficient placental implantation, with multisystem consequences (generalized endotheliosis) in the mother (Roberts and Hubel, 2009). Its incidence in industrializedcountries ranges from 3 to 5% (Stone et al., 1995; Dahlstrom et al., 2006; Wallis et al., 2008), which reaches up to 16% in Nigeria (Osungbade and Ige, 2011). The frequency of maternal and perinatal deaths in Mexico is 34 and 33%, respectively (Peralta Pedrero et al., 2006).

Preeclampsia is classified as mild and severe and can complicate to hemolysis, elevated liver enzymes, and low platelet count (HELLP) syndrome and eclampsia (Geller et al., 2004).

The pathophysiology of preeclampsia is characterized by placental hypoxia and/or ischemia leading to overexpression of hypoxia-inducible factor 1 (HIF1), which in turn increases the expression of the soluble isoform of vascular endothelial growth factor (sFlt) (Nevo et al., 2006). Hypoxic placenta also releases the soluble endoglin, antagonizing the production of endothelial nitric oxide synthase (eNOS) through sequestering of transforming growth factor beta1 (TGFb1) (Sandrim et al., 2008). In addition, the proangiogenic factors such as the placental growth factor (PIGF) and vascular endothelial growth factor (VEGF) (Reuvekamp et al., 1999). Together, they can trigger the antiangiogenic state in the mother, which resulted in generalized endothelial dysfunction.

Risk factors for disease development include nulliparity, pregnancy with multiple products, previous history of preeclampsia, vascular and connective tissue diseases, maternal age of >35 years, Afro-American ethnicity, preexistent renal disease, arterial hypertension, type 2 diabetes, and obesity (Eiland et al., 2012).

Previous studies on preeclampsia have considered the mother’s participation almost exclusively. However, the placenta is a transient biparental organ with maternal and paternal contributions. In this way, changes on the expression profile of genes involved in the metabolism and transport in the placenta depend on various maternal and paternal genetic profiles (Zusterzeel et al., 2002; Dekker et al., 2011).

Increasing evidence has demonstrated the father’s role in the onset of preeclampsia. In Astin et al. (1981) reported a case of a man who fathered two consecutive women who developed severe preeclampsia and passed away, suggesting the existence of a “fatal father factor.”

The risk of preeclampsia is genetically attributable to the mother (35%), fetus (20%), and couple (13%) (Cnattingius et al., 2004). Women and men who were born from a pregnancy with preeclampsia are at higher risk to have a baby, result of a preeclamptic pregnancy (Esplin et al., 2001). The change of partner increases the risk for preeclampsia in 1.6%, which increases up to 2.9% in a woman whose second pregnancy is the result of union with a man who has had a previous partner with preeclampsia (Lie et al., 1998). Change of partner plays a predisposing or protective role, depending of the presence or absence of the disease in the first pregnancy (Wikström et al., 2012). The presence of fetal variants of HLA-G from the father and those outside the mother generate a paternal-fetal susceptibility component for the development of preeclampsia (Tan et al., 2008).

Paternal genetic material plays an important role for the onset of preeclampsia. A triploid (69 XXX) partial mole, with paternal isodisomy in the placenta and a disomic fetus, increases preeclampsia-like symptoms at 19 weeks of gestation (Yoneda et al., 2013). Although preeclampsia is a human-specific disorder, animal models of the disease have been created to show the role of the paternal genetic factors. A female transgenic mouse for human angiotensinogen gene was mated with a male transgenic mouse for human renin gene, originating a preeclampsia-like syndrome (Takimoto et al., 1996).

In this review, we aimed to describe different lines of evidence regarding the paternal contribution on preeclampsia.

## Epidemiological and Clinical Evidence

Preeclampsia has been traditionally considered as a disease during first pregnancies; however, an early study by Need (1975) showed that a woman, pregnant by different fathers, had a healthy twin pregnancy with the first men, but severe preeclampsia occurred in the second one. The analysis of 34,201 patients revealed the presence of 47 multigravid patients with severe preeclampsia. A new partner was demonstrated in 13 among them (19.1%), and significant difference was observed than those in the control group (*p* < 0.01) (Feeney and Scott, 1980). Ikedife (1980) showed that change of partners was observed in 34 (74%) out of 46 multiparous patients with eclampsia. A woman with three different husbands presented preeclampsia only in the first pregnancy of the second and third spouses. Therefore, preeclampsia is not just a primiparous woman disease but also a condition related to the first pregnancy with a particular partner (Chng, 1982). The Guadeloupe study revealed an increased change of partner in multiparous women who were affected with preeclampsia (Robillard et al., 1993). The same group demonstrated that the incidence of preeclampsia was 11.9% among primigravidae, 4% among multigravidae without a change of paternity, and 24% among multigravidae with a new partner; however, these numbers depend on the duration of sexual cohabitation before conception (Robillard et al., 1994).

When evaluating paternal vs. maternal half-sisters with preeclampsia, Lie et al. (1998) found an increased risk for the disease in the former. Likewise, they showed that 13% of primiparous mothers with preeclampsia had recurrence in the second pregnancy, decreasing the influence of partner’s change (11.8%), which is opposite in mothers without preeclampsia in their first pregnancy (Lie et al., 1998).

The same results were obtained by Li and Wi (2000). Pipkin (2001) observed that partner’s change increases the risk for preeclampsia occurrence in primiparous women.

A woman pregnant by a partner who previously fathered a woman with preeclampsia was highly at risk to develop the disease. In 2004, Cnatinggius et al. evaluated three different scenarios: (1) mothers without preeclampsia and no partner change (control group); (2) mothers with previous preeclampsia, current preeclampsia, and change of spouse who was not fathered by a previous couple with preeclampsia; and (3) mothers with preeclampsia and change of couple who fathered another woman with preeclampsia. The maternal effect was 45%, meanwhile the paternal one was 10% (Cnattingius et al., 2004). Conversely, Chigbu et al. (2009) evaluated two groups of Nigerian women with and without change of partner in their second pregnancy and found no differences for the development of preeclampsia.

Marti and Herrmann (1977) found that preeclampsia is associated with shorter exposure to spermatozoa in younger women and more frequent use of barrier contraceptive methods. Therefore, the authors coined the term “immunogestosis” to denote the immune nature of preeclampsia. The use of barrier contraceptive methods was significantly higher in women with preeclampsia compared to healthy women [odds ratio (OR) = 2.48], and the number of sexual contacts was inversely related to the risk of preeclampsia (Klonoff-Cohen et al., 1989). Conversely, Mills et al. (1991) and Ness et al. (2004) found no differences in the use of barrier contraceptive methods between women with and without preeclampsia.

Shorter sexual intercourse increases the shorter antigenic seminal exposure (Beer, 1989). The risk of pregnancy-induced hypertension (PIH) was increased when conception is within 12 months of sexual cohabitation: 40% in 0–4 months, 23% in 5–8 months, 15% in 9–12 months, and 5% after 12 months (Robillard et al., 1994). The same author compared patients with simple PIH versus those with preeclampsia and eclampsia and observed that the sexual cohabitation times are shorter (9.5 months) compared to those of unaffected women (26.3 months) (Robillard and Hulsey, 1996). Koelman et al. (2000) found a lower frequency of oral sex in women with preeclampsia compared to healthy pregnant women (*p* = 0.0003), suggesting a protective role. The authors report lower amounts of soluble HLA A and HLA B in the seminal fluid, in partners of preeclamptic women (Koelman et al., 2000). In Robertson et al. (2003) proposed how repeated semen exposure protects preeclampsia, based on four lines of evidence: (1) the semen contains antigens shared by the conception; (2) after the seminal contact, the maternal mucosa can mount a regulated immune response to semen antigens; (3) semen contains among others, high amounts of TGFb that can inhibit type 1 immunity; and (4) TGFb-dependent changes in T-lymphocytes allow a hypo-responsiveness to paternal antigens. The exposure to seminal fluid through the vagina is inversely correlated with the risk of preeclampsia occurrence, whereas the oral exposure to seminal fluid has no effect on disease development (Saftlas et al., 2014).

A clear increase in the frequency of preeclampsia when the father (OR = 2.1) and mother (OR = 3.3) are products of preeclampsia-complicated pregnancies has been observed; however, this study did not consider the changes of paternity (Conde-Agudelo, 2001). In men who were born after a pregnancy complicated by preeclampsia, the risk in the first pregnancy was moderately increased compared with men who were born after a pregnancy without preeclampsia (OR = 1.5, 1.3–1.7).

This observed risk increases when severe or early preeclampsia is considered (OR = 1.9, 1.4–2.5) (Skjaerven et al., 2005). Lie (2007) found similar numbers: fathers who came from a preeclamptic pregnancy had a 1.5-fold risk [95% CI 1.3–1.7] of fathering a preeclamptic pregnancy.

Since, Need et al. (1983) reported a significantly higher frequency of preeclampsia in women with abortions (15.7%) than those with normal pregnancy (4.7%). They also found that azoospermia and oligospermia present a lower frequency of preeclampsia of 8.7 and 13.6%, respectively, which could be related to the minimal antigenic sperm exposure. The frequency of preeclampsia was higher in the donor insemination program than those in the father insemination program (OR = 1.20, 95%) (Smith et al., 1997). Hoy et al. (1999) revealed a higher frequency of preeclampsia in donor-inseminated women vs. natural-inseminated women (OR = 1.4, 95% CI). In Salha et al. (1999) compared 72 infertile women subjected to sperm, ovum, or embryo donation, with the same number of pregnant women through insemination with their own ovum or partner’s spermatozoa (control group). Fourteen patients developed preeclampsia, 13 of whom belong to the group of donated gametes and 1 to the control group. Kyrou et al. (2010) found a marginally significant (*p* = 0.05) higher frequency of preeclampsia in women conceiving by a sperm donor compared to partners in spermatozoa insemination. The type of spermatozoa and number of previous insemination cycles were the variables that influenced the risk of preeclampsia (*p* = 0.012); in fact, the authors observed that the fewer the number of the insemination cycles, the higher the risk of preeclampsia (Kyrou et al., 2010). A recent meta-analysis of seven studies showed the association with preeclampsia in women conceiving with donor sperm (OR = 1.63) (González-Comadran et al., 2014). Another study evaluated the risk of preeclampsia in infertile women subjected only to sperm donation via intrauterine insemination (IUI) or *in vitro* fertilization (IVF), compared to those with primary sperm donation (IUI or IVF) followed by egg donation. A higher frequency of preeclampsia in the latter was observed; therefore, the authors conclude that double gamete donation is associated with increased risk for preeclampsia (Bartal et al., 2018).

The aforementioned studies support the theory of the immunological basis of preeclampsia.

## Immunological Evidence

Preeclampsia is a state in which alloantigen (placenta of paternal origin) must be recognized to avoid rejection (Saito et al., 2007). Trophoblast cells must express paternal alloantigens that must be recognized by the mother’s immune system. Extravillous trophoblast express different HLA-C, E, F, and G (Hackmon et al., 2017). HLA-C is the ligand of immunoglobulin-like receptors (KIR) that are expressed in decidual natural killer (NK) cells (Sharkey et al., 2008). HLA-C and KIR are polymorphic; therefore, many maternal/paternal different combinations are possible. Two KIR haplotypes exist A and B, with the latter stimulating the expression of chemokines and angiogenic cytokines, promoting trophoblast invasiveness. Therefore, haplotype B could be protective for preeclampsia (Redman and Sargent, 2010). Seminal priming triggers a cascade of events for placental recognition or rejection. Seminal fluid contains high amounts of TGF-b that induces T-regulatory cells (Treg); these cells modulate immune responses in an antigen-specific way. Therefore, the effects of HLA-C/KIR interaction plus the seminal priming activity of TGF-b could in some extent explain the immune nature of father’s involvement in preeclampsia (Redman and Sargent, 2010).

The immune nature of preeclampsia was observed by Need (1975), who demonstrated histoincompatibility in a mother who developed preeclampsia with her second partner through the evaluation of HL-A typing. In Feeney et al. (1977) found a lower incidence of preeclampsia in previously blood-transfused women, compared with the same number of non-transfused primigravidas. A similar effect is observed in patients with kidney transplantation (Feeney et al., 1977). In the same year, Marti and Herrmann, (1977) found a correlation between the number of exposures to semen and lower frequency of preeclampsia; they coined the term immunogestosis to explain both, the immunologic tolerance and immunologic enhancement that abrogates immunoreaction against paternal and fetal histocompatibility antigens.

Birkeland and Kristofferson (1979) evaluated the immune response in mothers with and without preeclampsia and found no leukocyte antigens against the father in women with normal pregnancies, meanwhile these antigens were identified in one woman with severe preeclampsia. The evaluation of HLA A, B, and DR in women with severe and mild preeclampsia as well as their husbands and babies revealed a higher frequency of DR4 in all family members in severe preeclampsia (Kilpatrick et al., 1987). However, HLA A, B, and C are expressed in low amounts in the trophoblast, and HLA-G protein is exclusively expressed in trophoblast cells in high amounts. The evaluation of 1597del/C allele was not associated with preeclampsia (Aldrich et al., 2000). Three polymorphisms in HLA-G were evaluated in 68 primigravida trios, but were not associated with preeclampsia (Bermingham et al., 2000). The evaluation of 15 alleles in 4 exons of HLA-G in 155 family triads showed an overrepresentation of a homozygous HLA-G genotype in 40 pre-eclamptic offspring compared to 70 controls (*p* = 0.002) among primiparous women; further analyses suggested that the differences between pre-eclamptic cases and controls were primarily accomplished by a different transmission from the father of a 14 bp deletion/insertion polymorphism in the 3′UTR region (14 bp del/in) (*p* = 0.006) (Hylenius et al., 2004). In Tan et al. (2008) observed a significant association between paternally inherited HLA-G allele G^∗^0106 in the fetus and an increased risk for preeclampsia, but only in multigravid pregnancies. The 14 bp del/in was evaluated in three different combinations: mother/offspring, father/offspring, and couples; heterozygosity in the mother plus double insertion in babies was significantly higher in severe early-onset preeclampsia (*p* = 0.023), and the frequency of double deletion in both the father and baby was lower in severe early-onset preeclampsia (*p* = 0.024). The analysis of couples did not reveal significant differences between cases and controls (Zhang et al., 2012). A recent meta-analysis of 1,625 cases and 2,145 controls in all members of the triads evaluated the influence of 14 bp del/in in the onset of preeclampsia; the results did not reveal the association with the disease in offspring, mothers, or fathers. A stratification showed the association of 14 bp del/in with preeclampsia in European Caucasian offspring, but not found in African descent population (Pabalan et al., 2015).

Semen is not sterile, and its microbes have also been considered as a potential cause of preeclampsia. Repeated exposure to semen seems to create a memory protecting women from preeclampsia and the same could be possible in the case of semen microbes. Therefore, common elements of preeclampsia and infections such as Galectin13, Toll-like receptors, and antiphospholipid syndrome are found (Kenny and Kell, 2018).

## Genetic Evidence

The genetic nature of preeclampsia has also been evaluated through (1) familial cases, (2) twin studies, (3) consanguinity studies, (4) candidate gene evaluation, and (5) linkage analysis. Genes are involved in different pathophysiological mechanisms involved in preeclampsia. Genetic studies that included mother/father/children triads, which allow to determine the sole or joint contribution for preeclampsia, were limited.

The evaluation of thrombophilic genes methyle netetrahydrofolate reductase *(MTHFR)* and *FVL* in 92 mother/father/child triads revealed an increased risk only in mothers carrying two mutated copies of *MTHFR* and one mutated allele in *FVL*. Therefore, the risk of preeclampsia is not increased in the presence of fetal *MTHFR* or *FVL* mutations (Vefring et al., 2004). In van Dijk et al. (2010) observed a methylated paternal copy of *STOX1* gene and an unmethylated active maternal copy of the gene. Maternal transmission of this gene has been demonstrated in preeclampsia (van Dijk and Oudejans, 2011). The evaluation of *GSTP1, eNOS*, and *LPL* genes in 167 preeclamptic and control triads in a Greek cohort, which significantly demonstrated higher frequencies of Val105 allele (*GSTP1*) and Glu298Asp (*eNOS*) in control vs. preeclamptic groups in mothers, fathers, and child. The -93 polymorphism (*LDL*) was higher in preeclampsia only in mothers, but not in fathers or children. The transmission disequilibrium test revealed no differences in the rate of transmission of the studied common vs. mutated alleles (Pappa et al., 2011).

In Zhou et al. (2013) found a significant association of C4599A polymorphism in *AGTR2* gene with preeclampsia in mothers with body mass index (BMI) of ≥25 kg/m^2^ in their partners and studied children. Galaviz-Hernandez et al. (2016) evaluated the polymorphism rs5370 of *EDN1* gene in mothers with preeclampsia and their partners, showing a significant negative association with the disease in case of fathers (OR = 0.42; CI 95%, 0.18–0.94, *p* = 0.034), which was strengthened after adjusting the paternal protective factors. The evaluation of two polymorphisms in *SOD1* (+35A/C) and *SOD2* (Ala16Val (C/T) genes in 698 mother/father/infant triads revealed a significant association with preeclampsia in fathers with Ala16Val (TT genotype) [OR = 2.77 (1.32–5.81), *p* = 0.007]. This study revealed essentially the same risk for preeclampsia in both combined TT genotypes in mothers and fathers [OR = 6.80 (2.32–19.95), *p* < 0.001] and mother/father/infant triads [OR = 6.46 (2.16–19.31), *p* < 0.001] (Luo et al., 2018). Polymorphisms in the thrombophilic genes factor V Leiden, prothrombin, and *MTHFR* were evaluated in women, fetus, and fathers as risk factors for pregnancy-associated complications, including preeclampsia. The authors found significant differences between cases and controls in maternal Factor V and fetal *MTHFR*, but no differences on any of the polymorphisms analyzed in the father’s group (Nevalainen et al., 2018).

## Conclusion and Perspectives

Epidemiological, clinical, immunological, and genetic evidences supported the contribution of fathers in the onset of preeclampsia. Despite this, few studies were intended to evaluate the spouses’ role. In this way, the evaluation of paternal-derived immune and genetic materials was performed in order to identify the risk and prognostic markers of preeclampsia. Recent advances in placental epigenetics, along with the use of OMICS tools, ensure the identification of molecular markers associated with the role of fathers in the development of preeclampsia.

## Author Contributions

CG-H gathered and analyzed the data, and wrote the manuscript. MS-M gathered and organized the data and references. ET organized the data. MS-M, JG-O, and ET critically lectured the manuscript. BL-R gathered the data.

## Conflict of Interest Statement

The authors declare that the research was conducted in the absence of any commercial or financial relationships that could be construed as a potential conflict of interest.
